# 

**Published:** 1890-07

**Authors:** 


### CONTENTS, VOL. VI.

Amœba Coli, Observations on. .......................... 419
A New Medical College Proposed.......................... 139
Abstract Address by Dr. Howeth.......................... 288
Aneurism Plantar Arch.................................. 297
Asepsis vs. Antisepsis in Accidental Wounds............... 299
Appendicitis........................................... 238
Antipyretics, Improper use of........ .................... 434
Acute Hypertrophy Mammary Glands ....................... 497
Apocynum Cannabinum.................................. 505
Book Notices..................41, 92, 131, 217, 273, 320, 418, 484
Dysentery.............................................. 13
Duty (the) of Texas Legislature on Sanitation............... 337
Discussion on Abortion, by Medico-Legal Society, Chicago.. 383
Death (Sudden) During Labor ........................... 197
Carcinoma of Stomach ................................. 384
Cæsarean Section ..................................... 387
Cystic Tumor Inferior Maxilla........................... 97
Cancrum Oris.......................................... 440
Cholera Infantum, Ice Water in......................... 198
Clinical Notes From Practice.................... ....... 493

CORRESPONDENCE.

Letter From Dr. E. J. Beall, his trip to Europe.............. 143
Letter From Same—London ............................. 113
Letter from Dr. S. H. Stout, Archives of the Medical Department, C. S. A. ...................... ................ 68
Letter From Dr. H. K. Leake, from Birmingham............ 158
Letter From Dr. Jas. Johnson, on Unjust Treatment of Physicians by the State.......................... ......... 203
Extra Uterine Pregnancy, 4 months, delivered per Vias Naturalis............................................ 325
Ectopic Gestation, Indications for Operation.............. 246
International Medical Temperance Conference............. 507

EDITORIALS.

Asylum Superintendents appointed.................... 316
A Pull Altogether.................................... 177
A Plan Suggested .................................... 123
Captal Punishment; the Kremler Case............ .... 83
Death of Dr. Underhill................................ 301
Dr. Dock's Article .................................... 451
Doctors and "Doctors"................................ 27
Dastardly Outrage on a Physician...................... 401
Dr. Ghent's Hepatic Pills—"dose 1 to 3"............... 257
Finances of Texas State Medical Association........... 79
Homeopaths and Homeopathy under the Calcium Light 389
Hope Deferred ...................................... 404
Injustice to Doctors by the State..................... 213
Koch's Cure......................................... 259
Medical Bill, The.................................... 356

Medical Legislation and the Quarantine Bill............ 449
Necessity (the) for a State Board of Health............ 313
"Public Health Public Wealth"...................... 215
Quarantine Bill, The..... ........................... 33
Quacks in and out of the Profession.................. 449
Small Pox in Texas. ................................ 119
Senators in Motley................ ................ 313
Texas State Medical Association...................... 398
The Coming Operation for Cataract Extraction......... 178
The Waco Meeting, Texas State Medical Association .. 33
Transactions Texas State Medical Association for 1890. 180
Vale............................................. 491
Was it Consideration for Good of Inmates or was it
Politics?......................................... 125
State Lunatic Asylum at Austin ...................... 573
The Great Medical Temperance Movement............ 518
The "Decline and Fall Off"...................... ..... 520
"Our Noble Profession"............................ 516
Fibro Cystoma, Laparotomy for.......................... 107
Fracture Head of Humerus............................ .. 231
Gastro-Enterostomy...................................... 22
Gunshot Wound of Abdomen......................... .. 110
Hermaphrodite (a) in Lunatic Asylum.................... 236
Hæmatinuria vs. Malarial Hæmaturia..................... 438
Improper Use of Antipyretics............................. 434
Limitations of the Healing Art............................ 1
Malarial Hæmaturia...................................... 192
Medical Legislation, Quarantine Bill...................... 443
Medical News and Miscellany, 361, 35, 452, 182, 176, 142, 317, 86, 118, 127, 261, 412, 447, 321, 527.
Pedicle, Management of in Ovarian Cystoma ............. 432
Plantar Neuritis and Resection of P. Nerve ............... 340
Psoas Abscess Mistaken for Hernia .. .................... 58
Preliminary Capsulotomy in Cataract Extraction ........... 189
Proceedings Texas State Medical Association at Waco ..... 457
Perineum, Complete Rupture of.......................... 341
Resections on Alimentary Canal ......................... 275
Remarkable Suicide of an Insane Man.................... 56
Remarkable Case of Desqumation........................ 249
Small-Pox; Vaccination................................... 330
Septicæmia.............................. ............. 51
Surgical Clinic of Professor Rogers ...................... 334
Tracheotomy for Removal Safety Pin in Larynx ........... 112
Tubercular Peritonitis with Ascites Following Measles...... 245
Transplantation of Tissue from Lower Animals to Man..... 373
Traumatic Erysipelas Aborted............................ 200
Tracheo-Laryngotomy.................................... 441
Society Notes 406, 410, 250–1–2, 116–7, 352, 166–76, 448, 72, 177, 205–7–9, 509.
Selections...............................349, 305, 253, 210, 522
Urethral Caruncle, Treatment............................ 503

Three cases of malignant diphtheria—one death—occurred in a family six miles from Austin, in Dr. Morris' practice. No possible connection can be traced to any infected person or thing; and no local cause can be found. The water is from a clear, gushing mountain spring; what can be the cause?

---

Death of Dr. Towsey.—Dr. Samuel A. Towsey, one of the old "land marks" of Galveston, died in that city on the 11th inst. He was born in England in 1810; studied medicine and graduated in that country, and came to Texas in 1837, and entered the Texas navy, where he served several years. He had resided in Galveston upwards of fifty years.

Death of Dr. S. T. Lowry, of San Antonio.—Died, on June 30, ult. near Bandera, Texas, Dr. S. T. Lowry, of San Antonio, Texas, aged 44 years.

Dr. Sylvanus Todd Lowry was born at Elkhorn, in Kentucky. There he practiced medicine with his father a short time, and removed to Texas, settling in San Antonio, about nine years ago. He was a graduate of Jefferson Medical College. He was a member of the Texas State Medical Association, and had contributed liberally to the medical literature of Texas. For some time past his health had been failing, in consequence of laborious practice, and at the advice of friends, he concluded to take an overland trip across the mountains of West Texas, in hopes of recuperating his strength. At Bandera he was attacked with acute peritonitis, which rapidly proved fatal. He leaves a wife and several children, and a large host of attached friends to mourn his untimely loss. The remains were sent to his old Kentucky home for interment. The West Texas District Medical Association attended the funeral in a body.

### Book Notices.

MEDICAL NEWS AND MISCELLANY—

Deaths, Removals, Marriages, Society Notes, Therapeutic Suggestions, etc., thirty paragraphs of news .. 35

BOOK NOTICES—

Orthopœdic Surgery; Wm. Wood & Co.; Keating's Cyclopedia, Vol. III.—Lippincott. ..... 41

PUBLISHER'S NOTICES—

Or Therapeutic Hints ..... 44

---

The Education of Young Girls.—Those of our readers having young daughters to educate, would do well to send for a catalogue of St Mary's Academy, Austin, Texas. (See advertisement in this issue.) We speak from personal observation when we say that it is one of the very best institutions in America for the education and training of girls. Not only is the course of instruction thorough, but every attention in given to the manners and morals of the little ones entrusted to the care of the Sisters; and they are made to feel *at home*. We have seen some of the finest specimens of glorious young womanhood—physically, morally and intellectually—turned out from the Academy, and can heartily recommend it, especially for children who have been deprived of either parent. Refer to us.

F. E. DANIEL, Editor.

